# A Path Analysis of How Environmental Friendliness Shapes Social Connectedness, Attitudes Toward Aging, and QOL

**DOI:** 10.1093/geroni/igaf122.3279

**Published:** 2025-12-31

**Authors:** Shiau-Fang Chao, Jun-Rong Huang, Tzai-Hung Wen, Hsuanlei Shao

**Affiliations:** National Taiwan University, Taipei, Taipei, Taiwan (Republic of China); National Taiwan University, Taipei, Taipei, Taiwan (Republic of China); National Taiwan University, Taipei, Taipei, Taiwan (Republic of China); Taipei Medical University, Taipei City, Taipei, Taiwan (Republic of China)

## Abstract

**Background:**

The Ecological Model of Aging assumes that an individual’s aging experience is embedded within their living environment and influenced by community resources. This theoretical perspective suggests that environmentally friendly communities may improve older adults’ social connectedness, reinforce positive attitudes toward aging, and subsequently enhance their quality of life.

**Methods:**

This study employed path analysis to analyze data from 1,834 older adults drawn from the 2015 Taiwan Longitudinal Study on Aging.

**Findings:**

1.Direct effects: (1) All four environmental friendliness factors (housing satisfaction, medical treatment accessibility, perceived neighborhood safety, and environmental satisfaction) positively predicted higher social connectedness, as well as positive attitudes toward aging. (2) High social connectedness and positive attitudes toward aging were directly linked to older adults’ quality of life, with attitudes toward aging exhibiting the strongest direct effect. 2.

**Indirect effects:**

(1) Environmental friendliness factors led to enhanced social connectedness, which fostered positive attitudes toward aging, which ultimately improved quality of life. (2) Environmental friendliness factors also contributed to quality of life through their positive impact on attitudes toward aging, demonstrating the crucial mediating role of aging attitudes.

**Conclusions and Implications:**

Our findings highlight the significant role of environmental friendliness, social connectedness, and attitudes toward aging in determining older adults’ quality of life. Attitudes toward aging emerged as the strongest predictor of well-being. Environmental friendliness creates opportunities for social interaction, positively shaping aging perceptions and daily experiences. These results inform aging-in-place policies and community interventions that foster age-friendly environments supporting social connectedness and positive aging attitudes.

